# Geriatric Assessment Implementation before Chemotherapy in MEtastatic Prostate Cancer, Results from the Real-Life Study GAMERS

**DOI:** 10.3390/jcm12041636

**Published:** 2023-02-18

**Authors:** Cassandre Gluszak, Loïc Campion, Valérie Seegers, Oana Cojocarasu, Jean-Marie Commer, Frank Priou, Frédéric Rolland, Catherine Terret, Sophie Abadie-Lacourtoisie

**Affiliations:** 1 Department of Medical Oncology, Integrated Center of Oncology (ICO) Paul Papin, 49055 Angers, France; 2 Department of Biostatistics, Integrated Center of Oncology (ICO) René Gauducheau, 44800 Saint-Herblain, France; 3 Department of Biostatistics, Integrated Center of Oncology (ICO) Paul Papin, 49055 Angers, France; 4 Department of Medical Oncology, Centre Hospitalier Le Mans, 72037 Le Mans, France; 5 Department of Supportive Care, Integrated Center of Oncology (ICO) Paul Papin, 49055 Angers, France; 6 Department of Medical Oncology, Centre Hospitalier La Roche-sur-Yon, 85000 La Roche-sur-Yon, France; 7 Department of Medical Oncology, Integrated Center of Oncology (ICO) René Gauducheau, 44800 Saint-Herblain, France; 8 Department of Medical Oncology, Leon Berard Institute, 69008 Lyon, France

**Keywords:** geriatric assessment, implementation study, real-world, G8 screening, docetaxel, prostate cancer, older patients

## Abstract

Geriatric assessment (GA) can predict and improve treatment tolerance and estimate overall survival in older patients with cancer. Several international organizations promote GA; however, data related to its implementation in daily clinical practice are still limited. We aimed to describe GA implementation in patients over 75 years old with metastatic prostate cancer treated with docetaxel as first-line treatment, and with positive G8 screening test or frailty criteria. This retrospective real-world study included 224 patients treated from 2014 to 2021 in four French centers, including 131 patients with a theoretical indication of GA. Among the latter, 51 (38.9%) patients had GA. The main barriers to GA were the lack of systematic screening (32/80, 40.0%), unavailability of geriatric physician (20/80, 25.0%), and absence of referral despite a positive screening test (12/80, 15.0%). With GA performed in only one-third of the patients with a theoretical indication in daily clinical practice, mostly due to an absence of screening test, the use of GA is currently sub-optimal.

## 1. Introduction

With 70% of prostate cancer diagnosed in patients aged 70 and over, and 80% of disease related-deaths occurring in patients over 75 years of age, prostate cancer is a critical issue in geriatric medicine [1,2]. The standard first-line chemotherapy is docetaxel administrated every three weeks, or marginally adapted every two weeks, or every week [3,4]. 

Geriatric oncology arises from the need to address both oncologic and geriatric concerns and aims to better adjust treatment, and to prevent potential over- or under-treatment in older patients. 

Geriatric assessment (GA) consists of a comprehensive evaluation of older patients to support the therapeutic decision process [5]. GA also contributes to better assess prognosis [6] and treatment toxicity [7,8], to detect unknown geriatric problems [9], and to improve treatment tolerance [10,11].

However, GA is time-consuming and requires health care practitioners with skills in geriatrics and dedicated allocated time, oncologists familiar with screening tools such as G8, and specific patient pathways [12]. 

Several international organization promoted the use of GA [5,13,14] and its implementation in France was supported by the deployment of geriatric oncology coordination units (UCOG) throughout the country [15,16,17].

Several studies evaluated its implementation in a declarative way; however, data related to real world clinical practices are not available [18].

We conducted this multicentric retrospective study in patients aged 75 and over treated with docetaxel as first-line chemotherapy for a metastatic prostate cancer. 

The main goal was to evaluate the proportion of patients with a theoretical indication for GA (positive G8 screening test or frailty criteria) who effectively underwent GA evaluation. Reasons for non-assessment were collected.

We additionally assessed the routine use of screening tools by oncologists with the proportion of patients older than 75 years who received a G8 screening at the initial consultation.

## 2. Materials and Methods

### 2.1. Patients

Patients over 75 years old treated with docetaxel as first-line chemotherapy for a metastatic prostate cancer between 2014 and 2021 in four French care centers were included.

Study centers were French Comprehensive Cancer Centers (FCCCs) (n = 2) or hospitals of regional outreach (n = 2).

Eligible patients had histologically proven metastatic adenocarcinoma, hormone sensitive or refractory, with or without visceral metastasis. 

All patients were treated with docetaxel as first-line chemotherapy, every three weeks (standard regimen), or adjusted to every two weeks, or every week.

Patients opposed to the use of their data were not included. Patients with another proven histology, with concurrent prostate irradiation, or treated with a prior cytotoxic treatment were excluded.

### 2.2. Geriatric Evaluation

Theoretical indication of GA was defined by a positive screening test (G8 score ≤ 14), or by the presence of at least one of the following frailty criteria: 

Severe malnutrition (weight loss ≥ 10% in one month or 15% in the last six months or Body Mass Index <18 or albuminemia <30 g/L), Charlson comorbidity index ≥1, polypharmacy (≥5 drug classes per day), functional disability (IADL ≤ 3/4 or ADL ≤ 4/6), or neurocognitive impairment (subjectively assessed by the oncologist).

Geriatric assessment was conducted by general practitioners with skills in geriatrics or by a geriatrician depending on local possibility (no specific analyses regarding the kind of profession involved were performed).

This assessment explored comorbidities, polypharmacy, functional status, nutrition, geriatric syndromes (risk of fall and confusion, incontinence, neurosensory problems, etc.), social support, psychiatric health, and cognition.

### 2.3. Endpoint

The primary endpoint was the proportion of patients receiving a geriatric assessment, amongst those with theoretical indication. This rate was calculated in the total study population, and according to study centers and considering two recent four-year periods: 2014–2017 and 2018–2021.

A secondary endpoint was barriers limiting the GA; reasons for the absence of GA were extracted from electronic patient records.

We also determined the proportion of patients who had a G8 screening test completed. 

### 2.4. Statistical Analysis

Quantitative variables were described with mean, standard deviation, and range when clinically relevant and compared using student *t*-test. Categorical data were described using frequencies and percentage and compared using chi-squared or Fisher’s exact test, depending on the case. Statistical significance was fixed at *p* < 0.05. No missing data imputation was performed.

## 3. Results

### 3.1. Patients

From 2014 to 2021, data from 224 patients over 75 years old treated with docetaxel for metastatic prostate cancer in four study centers were collected.

**Figure 1:**
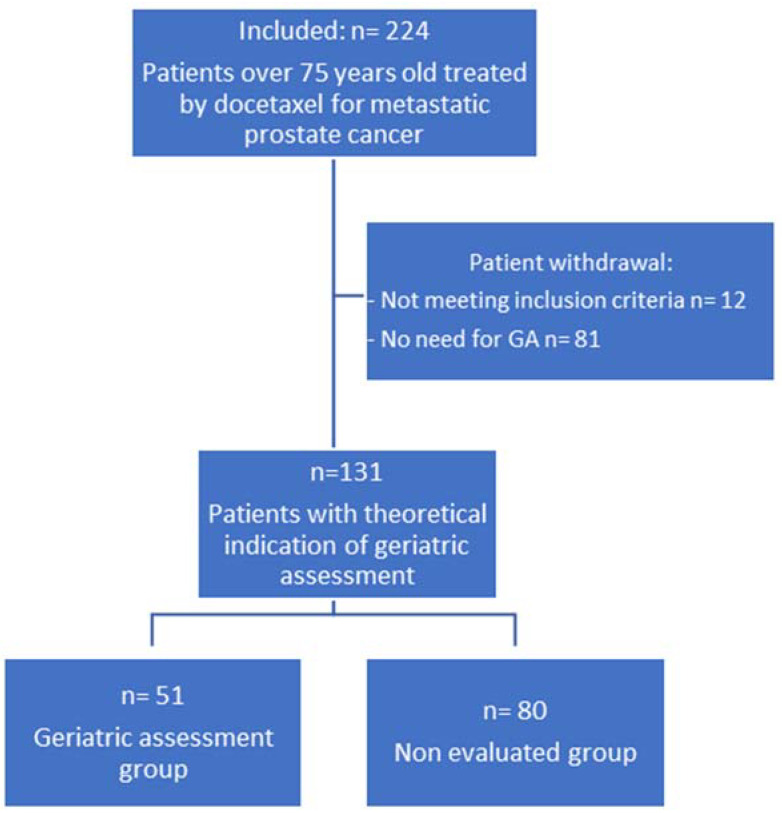
Patient flow diagram.

Among them, 131 had a theoretical indication for geriatric assessment, 51 completed GA and 80 did not complete GA (Figure 1).

Data from patients were collected in Angers (N = 82), in Le Mans (N = 10), in La Roche-sur-Yon (N = 16), and in Nantes (N = 23).

Patient characteristics based on geriatric assessment are summarized in Table 1. 

Patients with GA (N = 51) were older than patients in the non-evaluated group (N = 80) (81.4 ± 3.8 years vs. 79.4 ± 3.9 years; *p* = 0.006), with a higher proportion of patients over 80 years (33/51, 64.7%) vs. (34/80, 42.5%; *p* = 0.013).

Patients with GA were more likely to have an ECOG performance status (PS) ≥2 (35/51, 68.6% vs. 38/80, 47.5%; *p* = 0.018) or altered IADL (23/51, 45.1% vs. 13/80, 16.5%; *p* = 0.001). 

Patients with GA were more likely to have a Charlson Index ≥2, even if the difference was not statistically significant (20/51, 39.2% vs. 19/80, 23.8%, *p* = 0.059); mean CIRS scores (cumulative illness rating scale) were similar (9.9 ± 4.4 and 9.7 ± 4.6; *p* = 0.874). 

Polypharmacy was frequently observed, and reported in 40/51 (78.4%) patients with GA and 70/81 (87.5%) patients with no GA (*p* = 0.168), with a mean number of 8.3 ± 3.3 and 8.1 ± 2.6 medications per day, respectively.

Similar characteristics regarding tumor stage, volume, or hormonal status were observed.

### 3.2. Geriatric Assessment

One hundred and fourteen (50.9%) patients had a G8 screening test completed.

Significant variations in G8 completion were observed according to treatment centers (range 23.5–64.5%; *p* = 0.014). No significant improvement over time was reported (66/127, 52.0% from 2014 to 2017; 46/96, 47.9% from 2018 to 2021; *p* = 0.549)). 78 (68.4%); G8 screening tests were positive (G8 ≤ 14) (CI: 59.89–76.96).

In the patients with indication of GA, 51/131 (38.9%) patients had GA effectively performed. 

This proportion varied according to the study center (Nantes: 13/23, 56.5%; Angers: 37/84, 45.1%; La Roche-sur-Yon: 1/16, 6.3%; Le Mans 0/10, 0.0%; *p* < 0.001), but no significant differences were reported over time (2014–2017: 33/79, 41.8%; 2018–2021: 18/52, 34.6%; *p* = 0.411). The evaluation rate was higher in FCCCs with geriatrics trained practitioners onsite (Nantes and Angers) compared with hospitals with geriatrician in a distinct unit (La Roche-Sur-Yon and Le Mans).

**Figure 2:**
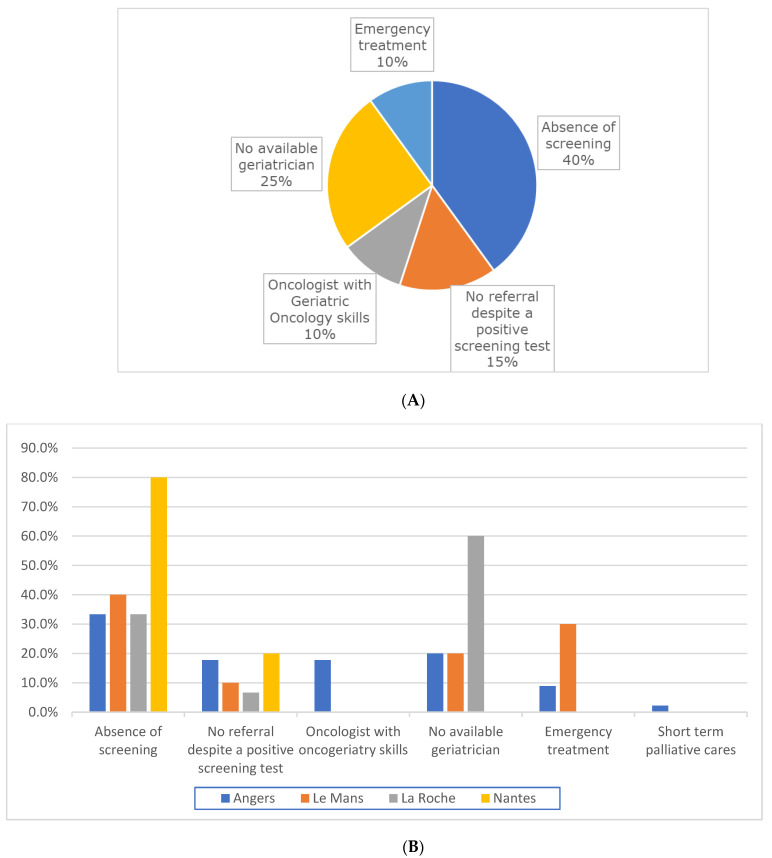
(**A**) Reasons for the absence of geriatric evaluation in the total population (N = 80). (**B**) Reasons for the absence of geriatric evaluation in each study center.

Reasons for the absence of geriatric evaluation are indicated in the total population (Figure 2A) and according to study sites (Figure 2B).

## 4. Discussion

French public health policy through the Plan Cancer “2003–2007” initiated the structuring of a geriatric oncology network and accredited 24 UCOGs, including five UCOGs with interregional duties. UCOGs aim to ensure local access to geriatric oncology care nationwide, to promote dedicated research, and to raise awareness and educate care providers.

The innovative study GAMERS evaluates geriatric assessment implementation in real-life practices in patients with prostate cancer in France. 

This study reported that GA implementation in real-life practice was achieved in only 38.9% of the eligible patients, with large variations reported according to the study center (range 0.0–56.5%). However, rates of GA implementation were stable over time.

GA feasibility and implementation in real practice was exclusively evaluated using questionnaires:

The ASCO Geriatric Oncology Task Force’s survey asked 1277 cancer providers about their current practice [18]. The use of a formalized GA was sub-optimal, which was consistent with results reported in the current GAMERS study: 29% of the clinicians reported the use of specific validated tools (ADL, IADL, Charlson comorbidity Index…), 69% reported an informal assessment based on their own judgment, and 57% of the practitioners reported that they “rarely or never” use validated tools to perform a multidimensional GA. 

In a survey including 332 cancer professionals, clinical practices in community cancer centers reported that only 17% of the physicians were performing GA whereas 95% agreed that it would benefit patients [19].

In these clinical settings, as opposed to the GAMERS study, patients were not referred to a geriatric physician and GA tools were instead used by the oncology team, which limits the generalization of the results in our system.

One of the mandatory steps in our GA process is screening using dedicated tools and performed by the oncologist, in order to identify patients at risk.

G8 score is the most frequently used in France, and is now mandatory in the tumor board report according to the French National Cancer Institute (INCA) guidelines.

This questionnaire is rapid (4.4 ± 2.8 min), reliable (sensitivity: 76.5%; specificity: 64.4%), and can be administrated by nurses in 87% of cases, according to the original publication [12]. 

Our results showed that its implementation in current practice is still insufficient, only being applied to 50.9% of patients. 

A nationwide Japanese survey reported similar findings from 32 specialized cancer centers: using mailed questionnaires, geriatric screening was achieved in 42% of the hospitals [20].

In this GAMERS study, the main reasons for non-assessment were absence of screening (40.0%), lack of available geriatric physician (25.0%), and absence of referral to a geriatrician despite a positive screening test (15.0%).

Major variation in unavailability of geriatric physicians was reported between study centers (0.0–60.0%). 

A U.S. study assessed the availability of geriatricians in 210 community oncology practices [21]: Geriatricians were available for consultation or co-management in 37% of the sites, with 13% of the sites having a geriatrician available to visit the patients in the oncology clinic, and 2% also had access to an onco-geriatrician. To the best of our knowledge, no similar data are available for France.

It is also interesting to report that 15.0% of the patients were not referred for GA, despite a positive screening score ranging from 10 to 14 and no other reason identified. 

Notably, the study ONCODAGE reported that the specificity of G8 test was 87.9% for prostate cancer with a threshold of 14, ensuring a low probability of false-positivity [12]. 

We did not investigate the reasons for failure in patient referral.

Barriers to GA implementation in daily practice have been explored in a declarative way. 

The ASCO survey, for example, reported barriers identified by oncologists [18]; organizational factors such as lack of time or limited clinical staff were the main limitations reported by clinicians aware of the ASCO guidelines, as well as the lack of training and awareness of the validated tools. 

Other practitioners were unaware of the ASCO guidelines; the main barriers mentioned were lack of training, knowledge, or awareness of the validated tools, and uncertainty regarding the most adequate tool to use.

A Dutch survey focused on obstacles reported by the geriatricians. Insufficient time or staff were the main barriers (43%), the lack of motivation in cancer specialists (36%), and the lack of prioritization within the geriatric department itself (26%) were also reported [22].

The GAMERS study reported a higher evaluation rate in FCCCs with geriatrics trained practitioners onsite (56.5% and 45.1%) compared with hospitals with geriatrician in a distinct unit (6.3% and 0.0% (*p* < 0.001)). 

Our study had several limitations, including the retrospective design and potential selection bias. In addition, data collected from patients included only four centers in the same administrative region. 

In addition, one of the inclusion criteria was neurocognitive impairment assessed subjectively by the oncologist, which is limiting. Interpretation needs to be cautious and generalizability of the results nationwide to all French cancer providers and practices is limited.

However, the GAMERS study is, to the best of our knowledge, the first study to assess real-world practices in terms of geriatric assessment accessibility in the field of prostate cancer, using data directly extracted from patient records. Our study design avoids bias related to declarative studies.

Several onco-geriatric models of care have been developed, but the gold standard still needs to be determined.

In clinical settings with no facilitated access to a geriatrician, GA could be either self-administrated or performed by nurses with skills in geriatrics and trained to use dedicated geriatric tools, and results could then be referred to an oncologist experienced in geriatrics for interpretation [23]. This model is the one recommended in ASCO guidelines.

An alternative model developed in the Netherlands relies on a primary nurse-led geriatric assessment using dedicated tools, and results are further discussed in an oncogeriatric multi-disciplinary team, including a geriatrician providing treatment recommendations [24]. Patients are then referred to a geriatrician only if further evaluations are needed for management or treatment decisions (which represents 13% of the patients referred versus around 60% when considering patients with a positive screening test with usual scores such as G8 [12]).

In France, the screening is completed by the cancer care provider and further referral for a GA is performed if needed. 

Considering the general increase of the older population, and a potential marked discrepancy with the required number of trained geriatricians, further adjustment and improvement of the organization is required to remain effective [25].

The results of the GAMERS study open up new perspectives: 

First, referral to a geriatrician when available should be encouraged, and better training of oncologists and better collaboration between geriatric and oncology services are required.

Second, there is a need for policy direction to promote the training and availability of more geriatricians.

## 5. Conclusions

The GAMERS study showed that a third of eligible patients actually completed a geriatric assessment in real-world practices mostly due to sub-optimal use of the screening tools.

Further research is needed to investigate clinical practices nationwide and to evaluate the most effective interventions to promote geriatric assessment, support treatment decision, and improve global health care management in older patients.

## Figures and Tables

**Table 1:** Patients characteristics at baseline.

	Geriatric Assessment		
	Yes	No	
	(n = 51)	(n = 80)	*p*-Value
**Age**			
Mean ± SD	81.4 ± 3.8	79.4 ± 3.9	0.006
≥80 years, n (%)	33 (64.7)	34 (42.5)	0.013
**Performance status**			
0–1, n (%)	16 (31.4)	42 (52.5)	0.018
≥2, n (%)	35 (68.6)	38 (47.5)	0.018
**Comorbidities**			
**CIRS**			
Mean ± SD	9.9 ± 4.4	9.77 ± 4.6	0.874
**Charlson Comorbidity Index**			
Mean ± SD	1.2 ± 1.5	0.75 ± 1.0	0.062
0–1, n (%)	31 (60.8)	61 (76.3)	0.059
≥2, n (%)	20 (39.2)	19 (23.8)	0.059
**Chronic heart failure, n (%)**	5 (9.8)	8 (10.0)	0.999
**Polypharmacy**			
Mean ± SD	8.25 ± 3.3	8.05 ± 2.6	0.709
>5, n (%)	40 (78.4)	70 (87.5)	0.168
**Prostate disease, n (%)**			
Low volume	2 (3.9)	2 (2.5)	0.642
High volume	49 (96.1)	78 (97.5)	0.642
Visceral metastasis	20 (39.2)	21 (26.3)	0.127
**PSA (ng/mL)**			
Median (range)	101 (0.5–1739)	74 (0.1–5000)	
Mean ± SD	263.3 (399.2)	323.8 (737.8)	0.545
**Hormonal status, n (%)**			
Hormone-sensitive	6 (11.8)	19 (23.8)	0.112
Hormone-refractory	45 (88.2)	61 (76.3)	0.112
**G8 screening**			
Available, n (%)	41 (80.4)	37 (46.3)	<0.001
Mean ± SD	10.5 ± 3.0	11.6 ± 2.6	0.066
**Cognitive troubles, n (%)**	10 (19.6)	4 (5.0)	0.017
**Severe undernutrition, n (%)**	13 (25.5)	14 (17.5)	0.27
**ADL score ≤ 4 /6, n (%)**	6 (11.7)	4 (5.0)	0.186
**IADL score ≤ 3/4, n (%)**	23 (45.1)	13 (16.5)	<0.001

CIRS: Cumulative illness rating scale; PSA: Prostate-specific antigen; ADL: Activities of daily living; IADL: Instrumental activities of daily living. *p*-values are based on chi-squared tests for categorical variables and t test for continuous variables.

## Data Availability

The data presented in this study are available on request from the corresponding author.
